# Flushing Strategies for Totally Implantable Venous Access Devices in Nurse‐Led Cancer Care: Exploratory Randomized Clinical Trial

**DOI:** 10.1002/nop2.70942

**Published:** 2026-10-16

**Authors:** Chi‐Jia Ho, Jason Chia‐Hsun Hsieh, Hui (Grace) Xu, Chieh‐Ching Yen, Hsuan‐Chih Kuo, Chih‐Ting Chou, Li‐Chin Chen, Bo‐Cyuan Wang

**Affiliations:** ^1^ Department of Nursing New Taipei City Municipal Tucheng Hospital (Built and Operated by Chang Gung Medical Foundation) New Taipei City Taiwan; ^2^ College of Medicine Chang Gung University Taoyuan City Taiwan; ^3^ Division of Oncology, Department of Internal Medicine Chang Gung Memorial Hospital, Linkou Taoyuan City Taiwan; ^4^ School of Medicine National Tsing Hua University Hsinchu City Taiwan; ^5^ School of Nursing Queensland University of Technology Brisbane Australia; ^6^ Department of Emergency Medicine New Taipei City Municipal Tucheng Hospital (Built and Operated by Chang Gung Medical Foundation) New Taipei City Taiwan; ^7^ Division of Hematology‐Oncology, Department of Internal Medicine New Taipei City Municipal Tucheng Hospital (Built and Operated by Chang Gung Medical Foundation) New Taipei City Taiwan; ^8^ Nursing Management Department of Administration Center Chang Gung Medical Foundation Taoyuan City Taiwan; ^9^ Department of Nursing Chang Gung University of Science and Technology Taoyuan City Taiwan

**Keywords:** catheter occlusion, clinical trial, nurse‐led care, oncology nursing, standardized catheter care, totally implantable venous access device

## Abstract

**Aim (s):**

To examine catheter‐related complications across different flushing strategies for totally implantable venous access devices when care is delivered through a standardized, nurse‐led, protocol‐driven framework.

**Design:**

A single‐center, prospective, exploratory, randomized, single‐blind, three‐arm parallel‐group clinical trial.

**Results:**

Among 192 participants, catheter‐related complications were rare across all groups. One occlusion occurred in the 10 mL saline group, no occlusions occurred in the 20 mL saline or heparin lock groups, minor bleeding occurred in three participants, and no catheter‐related bloodstream infections or severe adverse events were reported. The very low number of events precluded meaningful comparative inference between flushing strategies.

**Conclusion:**

Within a standardized, nurse‐led catheter care framework, complication rates were uniformly low regardless of flushing strategy. These exploratory findings do not establish equivalence or comparative effectiveness between flushing strategies.

**Implications for the Profession and/or Patient Care:**

For oncology nursing practice, these findings support continued emphasis on standardized, protocol‐driven catheter care, competency‐based nurse training, and adherence monitoring; they do not establish that saline can replace heparin.

**Impact:**

This study addresses uncertainty about catheter outcomes under different flushing strategies in standardized nursing care. The main finding was uniformly low complication rates across groups. The findings may inform oncology nursing practice, catheter care standardization, and future trial design.

**Reporting Method:**

This study was reported in accordance with the CONSORT 2025 statement and relevant extensions.

**No Patient or Public Contribution:**

Patients and members of the public were not involved in the design, conduct, analysis, interpretation, or preparation of this manuscript because the study evaluated a protocol‐driven inpatient catheter care procedure delivered within routine oncology nursing practice.

**Trial Registration:**

ClinicalTrials.gov: NCT05707936 (https://clinicaltrials.gov/study/NCT05707936)

## 1 Introduction

Totally implantable venous access devices (TIVADs), also known as port‐a‐caths, are widely used in oncology care to facilitate repeated intravenous therapy (Bradford et al. 2020). Maintaining catheter patency is essential for treatment continuity and patient safety. However, catheter occlusion remains a clinically relevant complication, and its reported incidence rate ranges from approximately 1%–6% (Ince et al. 2020; Liu et al. 2023; Milani et al. 2017; Velioğlu et al. 2019). Catheter occlusion can result in treatment interruption, additional procedures, increased health‐care utilization, and premature device removal, thereby compromising continuity of care in patients with cancer (Ince et al. 2020; Liu et al. 2023; Milani et al. 2017; Taxbro et al. 2019; Velioğlu et al. 2019).

The effectiveness of TIVADs depends on not only device‐related factors but also the quality and consistency of routine catheter care in clinical practice. Nurses play a central role in catheter maintenance and vascular access safety. Multiple factors contributing to catheter occlusion, such as flushing practices, chemotherapy administration, thrombotic events, and indwelling duration, are strongly associated with nursing care processes (Liu et al. 2023; Milani et al. 2017). Many of these factors are modifiable, indicating that how nursing care is organized and delivered substantially affects catheter outcomes.

At the study site, heparin lock remained the routine TIVAD maintenance practice when the trial was initiated and was therefore retained as the active comparator. However, heparin is associated with adverse events such as bleeding and thrombocytopenia and may increase medication burden and health‐care costs (Goossens et al. 2013; Onishi et al. 2016). Despite the routine use of flushing strategies, systematic reviews and meta‐analyses have not demonstrated consistent superiority of heparin over normal saline for maintaining catheter patency. The certainty of evidence remains limited because of heterogeneity in study design, clinical settings, and catheter care practices (Bradford et al. 2020; López‐Briz et al. 2018; Wu et al. 2021; Zhong et al. 2017).

## 2 Background

Most studies have compared flushing solutions from a pharmacological perspective and paid less attention to the clinical context in which catheter care is delivered. The international infusion therapy guidelines state that standardized, evidence‐based catheter care and adherence to recommended techniques are crucial for maintaining catheter patency (Nickel et al. 2024). However, catheter care practices still vary widely among clinical settings because of differences in training, protocol availability, workflow constraints, and organizational factors (Vilão et al. 2024). This variability may lead to inconsistent clinical outcomes and may partially explain the uncertainty in the current evidence base.

Optimizing catheter care requires attention to not only specific interventions but also the consistency and fidelity of care delivery (Abou‐Mrad et al. 2024; Clari et al. 2021). Contemporary health‐care systems increasingly emphasize reducing low‐value or unnecessary interventions and strengthening evidence‐based practices. The routine use of heparin in TIVAD maintenance may represent a modifiable practice if comparable catheter patency and complication rates can be achieved with standardized saline‐based care in a structured care model. Despite these considerations, randomized evidence evaluating flushing strategies in a standardized, nurse‐led, protocol‐driven catheter care framework remains limited. This gap is particularly relevant to oncology nursing because simplified and standardized catheter care may improve medication safety, reduce costs, enhance workflow efficiency, and support implementation across diverse health‐care settings.

Taken together, existing evidence indicates that TIVAD outcomes should be considered in relation to both the flushing solution and the care delivery context. Evaluating flushing strategies within a standardized nursing care framework may therefore provide preliminary evidence to inform future adequately powered studies.

## 3 The Study

### 3.1 Aim(s) and Objective

This exploratory randomized study aimed to evaluate TIVAD‐related complications in hospitalized patients with cancer when catheter care was delivered through a standardized, nurse‐led, protocol‐driven framework. Specifically, the study compared early catheter‐related outcomes across three flushing strategies: 10 mL normal saline, 20 mL normal saline, and heparin lock after each TIVAD use.

### 3.2 Technical Terminology Used to Describe the Aim

TIVADs refer to implantable venous ports used to support repeated intravenous therapy. In this study, standardized nurse‐led catheter care referred to a protocol‐driven care framework delivered by trained oncology nurses and included skin antisepsis, pulsatile flushing, positive‐pressure technique, adherence monitoring, and feedback. Heparin lock referred to 1000 units of heparin diluted in 10 mL saline. Normal saline flushing referred to flushing with either 10 mL or 20 mL of normal saline after each TIVAD use.

### 3.3 Primary, Secondary, or Other Objectives

The co‐primary objective was to evaluate TIVAD occlusion and catheter‐related bloodstream infection (CRBSI) across the three flushing groups. Bleeding complications were evaluated as secondary outcomes. Exploratory analyses were conducted to estimate absolute risk differences and corresponding 95% confidence intervals to inform the design of future adequately powered studies.

## 4 Methods/Methodology

### 4.1 Design

This prospective, randomized, single‐blind, three‐arm parallel‐group study was conducted in a real‐world inpatient oncology setting and incorporated pragmatic elements, including routine clinical workflows and clinically relevant outcomes. Protocol adherence was supported through standardized care procedures.

Participants were randomly assigned in a 1:1:1 ratio to receive flushing with 10 mL normal saline (NS10), 20 mL normal saline (NS20), or heparin lock (1000 units in 10 mL saline, H/L) after each TIVAD use. Block randomization (block size: 4) was generated using Random Allocation Software (version 1.0; Mahmood Saghaei, Department of Anaesthesia, Isfahan University of Medical Sciences, Isfahan, Iran) (Saghaei 2004). Allocation concealment was ensured using sequentially numbered, opaque, sealed envelopes prepared by an independent researcher. A trained nurse practitioner screened and enrolled participants and opened the envelopes sequentially after enrollment to determine group allocation. Participants and outcome adjudicators were blinded to group allocation. Because of the nature of the intervention, nurses delivering catheter care and the statistician performing the analyses were not blinded.

The study is reported in accordance with the Consolidated Standards of Reporting Trials (CONSORT) 2025 statement and relevant extensions (Hopewell et al. 2025). The outcome hierarchy was unchanged from the trial registration. The outcome‐surveillance approach was modified after trial commencement, as described in Section 4.9; no changes were made to the randomized flushing interventions.

### 4.2 Outcome Measures and Assessment Procedures

The co‐primary outcomes were TIVAD occlusion and CRBSI. Occlusion was defined as a persistent inability to aspirate blood or flush saline despite sequential corrective steps, including pulsatile flushing with a 10‐mL syringe, patient repositioning, and repeat aspiration testing. A standardized, nurse‐led troubleshooting algorithm was used to assess suspected occlusion (Figure 1). Suspected occlusion events were adjudicated by an independent attending physician who was blinded to group allocation.

**FIGURE 1:**
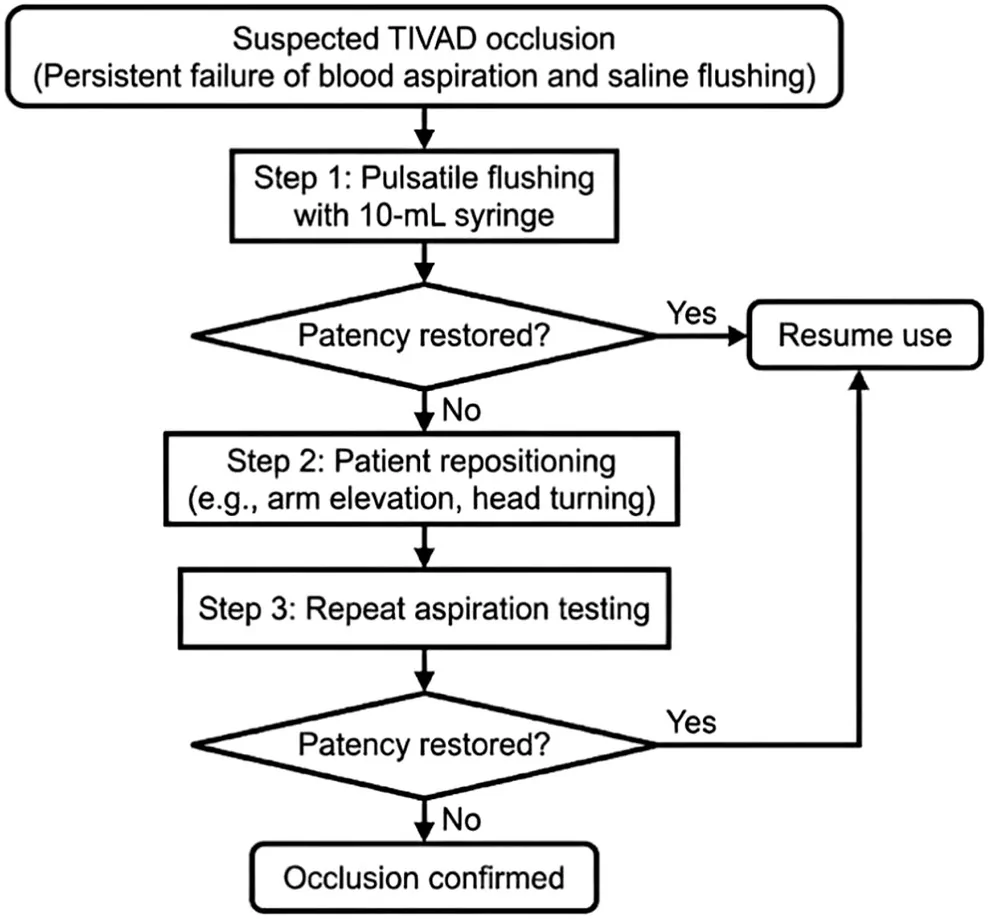
A nurse‐led, protocol‐driven troubleshooting algorithm for suspected TIVAD occlusion.

CRBSI was defined according to the criteria outlined by the Taiwan Center for Disease Control (Taiwan CDC 2018). Bleeding complications were evaluated as a secondary outcome and recorded when they required additional clinical or nursing intervention, including medication adjustment or compression.

### 4.3 Sampling and Recruitment

Eligible participants were screened by a trained nurse practitioner between February 1, 2023, and September 1, 2023, at a regional teaching hospital in northern Taiwan. The hospital provides comprehensive inpatient oncology care and implements routine nursing workflows and catheter management practices.

At screening, eligibility required an anticipated need for TIVAD use for ≥ 3 days during the index hospitalization to ensure adequate exposure to the assigned catheter‐care protocol. This criterion was determined before enrollment and randomization based on the expected course of inpatient treatment. All randomized participants were included in the intention‐to‐treat analysis.

### 4.4 Sample Size and Power

The sample size was estimated a priori by using G*Power (version 3.1.9.7; Heinrich Heine University Düsseldorf, Düsseldorf, Germany Faul et al. 2009, 2007) with *α* = 0.05 and power = 0.80. Because no established occlusion rate data specific to standardized care settings were available for precise sample size estimation based on proportional differences, a general ANOVA‐based approach with a moderate effect size (*f* = 0.25) was selected to allow feasible recruitment in the inpatient oncology setting (Rothwell et al. 2018). This calculation was therefore used pragmatically to establish a feasible recruitment target rather than to provide definitive power for detecting between‐group differences in the binary primary outcome.

Given the anticipated low event rates and the exploratory nature of the study, the trial was not powered to detect definitive differences in comparative effectiveness. Instead, the sample size was considered sufficient to generate preliminary data on safety, feasibility, and outcome patterns across groups. To account for potential attrition, the target sample size was increased by 20%, resulting in a final sample of 192 participants.

### 4.5 Quality Appraisal and Protocol Adherence

Quality assurance was addressed through standardized intervention delivery, nurse training, competency assessment, and protocol adherence monitoring. All oncology nurses delivering the intervention were trained and certified in accordance with institutional vascular access standards before the study. The training program included protocol education, skills demonstration, supervised practice, and competency assessment using a standardized checklist consistent with international infusion therapy standards. Refresher briefings were provided during the trial to reinforce adherence.

Adherence to the catheter care protocol was monitored through biweekly observational audits conducted by the head nurse using a structured checklist. The checklist assessed the correct execution of key procedural steps, including skin antisepsis, pulsatile flushing technique, and maintenance of positive pressure during the final flushing step. Feedback was provided during routine clinical meetings, and any observed deviations were addressed through reinforcement of the standardized procedure.

### 4.6 Data Abstraction

Baseline clinicodemographic characteristics, intervention allocation, TIVAD use information, and catheter‐related outcome data were abstracted from standardized study records and clinical records. Data abstraction followed predefined study variables and outcome definitions.

### 4.7 Population and Sample

The study population comprised hospitalized patients with cancer who had a TIVAD and were receiving intravenous therapy in oncology wards. All randomized participants were included in the intention‐to‐treat analysis.

### 4.8 Inclusion and/or Exclusion Criteria

The inclusion criteria were as follows: being aged ≥ 20 years, having a confirmed cancer diagnosis, having a TIVAD, receiving intravenous therapy during hospitalization in oncology wards, and having an anticipated need for TIVAD use for ≥ 3 days during the index hospitalization.

The exclusion criteria were as follows: having a history of catheter occlusion, receiving continuous intravenous infusion, such as parenteral nutrition, normal saline, or other maintenance fluids, receiving anticoagulant or antiplatelet therapy, having coagulation disorders, being pregnant, and using hormonal contraceptives.

### 4.9 Data Sources/Collection

The trial was initially registered with outcome assessment during the index admission, up to a maximum of 12 weeks. During early recruitment, the low overall occurrence of catheter‐related events prompted reconsideration of this admission‐based surveillance approach. Before any formal between‐group outcome analysis, surveillance was redefined as 30 days after enrollment or until device discontinuation, whichever occurred first. No group‐specific comparative outcome analysis informed this change. The 30‐day surveillance window was applied to all randomized participants, and complete outcome data within this window were available for all 192 participants. The revised approach provided a standardized early follow‐up window and allowed catheter‐related events occurring after discharge to be captured.

During the index hospitalization, participants received the assigned flushing protocol whenever the TIVAD was used. The randomized flushing intervention was limited to the index hospitalization; no study‐mandated flushing strategy was imposed after discharge. For participants discharged before completion of the 30‐day follow‐up period, outcome surveillance continued through review of routine outpatient and outpatient chemotherapy records.

A trained nurse practitioner screened eligible participants and recorded baseline and follow‐up data during the study period. Routine inpatient clinical records and subsequent outpatient records were screened for documentation or management suggestive of catheter‐related complications, and potential events were classified according to the predefined outcome definitions.

### 4.10 Data Analysis

Statistical analyses were performed using SPSS (version 29.0; IBM, Armonk, NY, USA). All analyses followed the intention‐to‐treat principle. Descriptive statistics were used to summarize baseline characteristics.

Continuous variables were analysed using one‐way analysis of variance or the Kruskal–Wallis test, as appropriate. Categorical variables were compared using Fisher's exact test. A two‐sided *p* value of < 0.05 was considered statistically significant. No data were missing; thus, no imputation was performed.

Given the low event rate, exploratory analyses were conducted to estimate absolute risk differences and corresponding 95% confidence intervals. These estimates were considered descriptive and hypothesis‐generating and were not intended to support confirmatory comparative‐effectiveness inference.

### 4.11 Ethical Considerations

The study protocol was approved by the relevant institutional review board (approval number: 202101793A3). This study adhered to the ethical principles of the Declaration of Helsinki. Written informed consent was obtained from all participants before enrollment. The trial was prospectively registered at ClinicalTrials.gov (identifier: NCT05707936).

## 5 Results

### 5.1 Subjects

The trial was completed as planned. A total of 192 participants were enrolled and randomized, with 64 participants allocated to each group. Figure 2 presents the CONSORT flow diagram. No participants withdrew from the study, and no outcome data were missing. No severe adverse events occurred during the study period.

**FIGURE 2:**
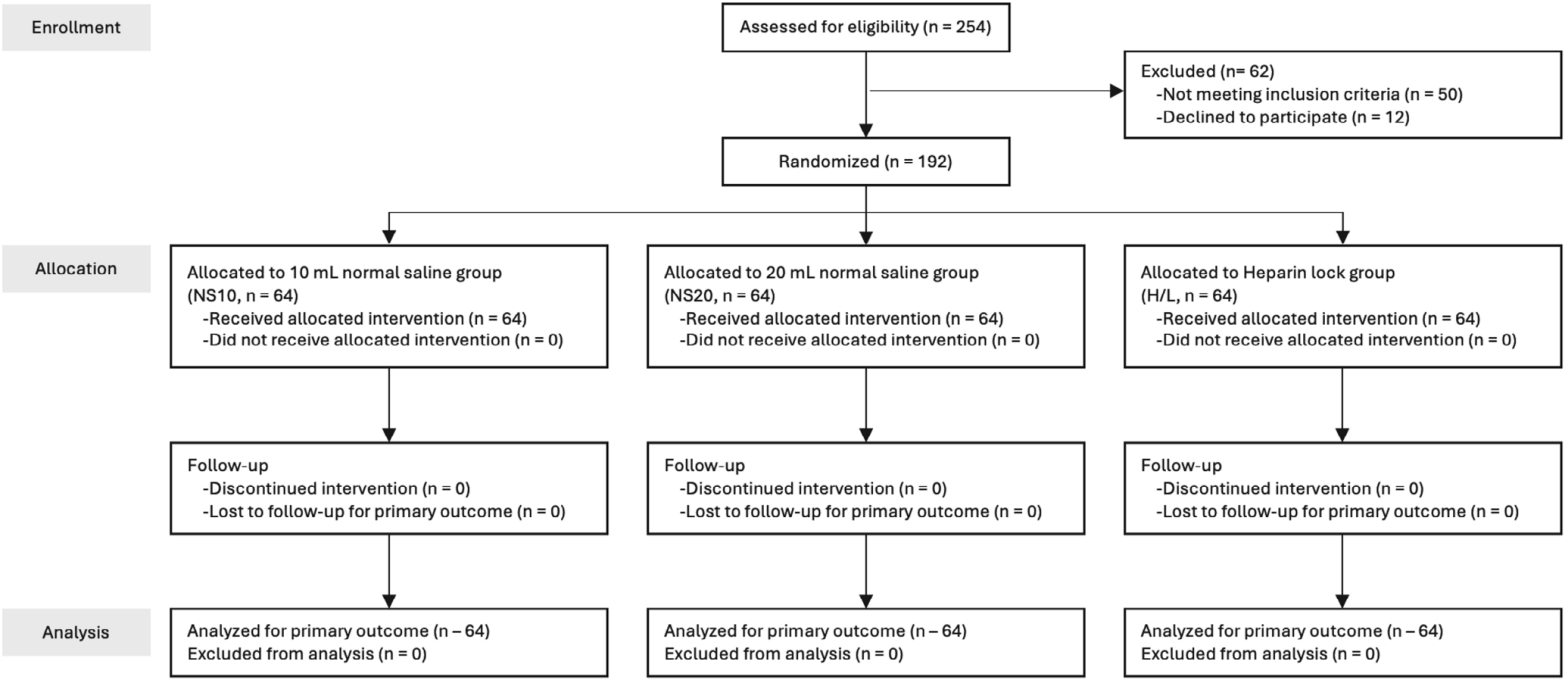
CONSORT flow diagram of participant screening, randomization, allocation, follow‐up, and analysis.

Baseline clinicodemographic characteristics were similar among the three groups (Table 1). The mean age was 58.01 ± 9.83 years, and sex distribution was balanced across the groups. The mean duration of exposure to the assigned flushing protocol during the index hospitalization was 7.13 ± 6.57 days. Gynaecological cancer was the most common malignancy (33.3%), followed by gastrointestinal cancer (26.0%), head and neck cancer (19.8%), lung cancer (12.5%), and other cancers (8.3%). No significant differences were observed among the groups in age, sex, cancer type, or duration of protocol exposure.

**TABLE 1:** Baseline clinicodemographic characteristics of the study groups.

Item	Total (*N* = 192)	Group			p*
		NS 10 (*n* = 64)	NS 20 (*n* = 64)	H/L (*n* = 64)	
Age (mean, SD)	58.01 ± 9.83	57.55 ± 10.46	58.02 ± 9.52	58.47 ± 9.62	0.870^a^
Sex					
Male	96 (50.0%)	34 (53.1%)	32 (50%)	30 (46.9%)	0.779^b^
Female	96 (50.0%)	30 (46.9%)	32 (50%)	34 (53.1%)	
Duration of exposure to the assigned flushing protocol during index hospitalization, days (mean, SD)	7.13 ± 6.57	6.80 ± 6.37	6.86 ± 7.00	7.72 ± 6.39	0.375^c^
Cancer type					
Gastrointestinal	50 (26.0%)	13 (20.3%)	20 (31.3%)	17 (26.6%)	0.596^b^
Gynaecological	64 (33.3%)	25 (39.1%)	21 (32.8%)	18 (28.1%)	
Head and neck	38 (19.8%)	16 (25.0%)	9 (14.1%)	13 (20.3%)	
Lung	24 (12.5%)	5 (7.8%)	9 (14.1%)	10 (15.6%)	
Others	16 (8.3%)	5 (7.8%)	5 (7.8%)	6 (9.4%)	

*Note:* Data are presented as *n* (%). Percentages for treatment groups were calculated using the column total as the denominator.

Abbreviations: H/L, heparin lock; NS10, 10 mL normal saline; NS20, 20 mL normal saline.

^a^ One‐way analysis of variance.

^b^ Chi‐square or Fisher's exact test.

^c^ Kruskal–Wallis test, as appropriate.

* *p* values were calculated using.

### 5.2 TIVAD Occlusion

All randomized participants were included in the co‐primary outcome analysis, and complete outcome data were available within the 30‐day surveillance window.

Catheter occlusion was rare, with only one event observed during the study period (1/192, 0.5%). The event occurred in the 10 mL normal saline group (1/64, 1.6%), whereas no occlusion events occurred in the 20 mL normal saline or heparin lock group (0/64, 0% in each group Table 2). Fisher's exact test yielded *p* = 1.000; however, the sparse event count precluded meaningful comparative inference between groups.

**TABLE 2:** Incidence of catheter occlusion, bleeding, and catheter‐related bloodstream infections in the study groups.

Item	Total	Group			p*
		NS10	NS20	H/L	
Occlusion	1/192, 0.5% (0.01–2.9)	1/64, 1.6% (0.04–8.4)	0/64, 0% (0–5.6)	0/64, 0% (0–5.6)	1.000
Bleeding	3/192, 1.6% (0.3–4.5)	2/64, 3.1% (0.4–10.8)	0/64, 0% (0–5.6)	1/64, 1.6% (0.04–8.4)	0.774
CRBSI	0/192, 0% (0–1.9)	0/64, 0% (0–5.6)	0/64, 0% (0–5.6)	0/64, 0% (0–5.6)	—

*Note:* Data are presented as *n*/N, % (95% CI). Confidence intervals for proportions were calculated using the exact binomial method.

Abbreviations: CRBSI, Catheter‐related bloodstream infections; H/L, heparin lock; NS10, 10 mL normal saline; NS20, 20 mL normal saline.

* *p* values were calculated using Fisher's exact test.

Exploratory analyses of absolute risk differences showed a risk difference of 0.0 percentage points between the 20 mL normal saline and heparin lock groups (95% CI: −5.7 to 5.7 percentage points) and 1.6 percentage points between the 10 mL normal saline and heparin lock groups (95% CI: −5.4 to 8.3 percentage points). Given the very low event rate, these estimates are imprecise and do not permit meaningful comparative inference between flushing strategies.

### 5.3 Catheter‐Related Bloodstream Infections and Bleeding

Bleeding events were infrequent, with three cases of minor bleeding, defined as localized bruising (ecchymosis) at the catheter site, observed during the study period (3/192, 1.6%). Two bleeding events occurred in the 10 mL normal saline group (2/64, 3.1%), one in the heparin lock group (1/64, 1.6%), and none in the 20 mL normal saline group (0/64, 0% Table 2). Fisher's exact test yielded *p* = 0.774; given the small number of bleeding events, this result was interpreted descriptively rather than as evidence of comparable bleeding risk.

No catheter‐related bloodstream infections, unplanned catheter removals, or device exchanges occurred in any group during follow‐up.

## 6 Discussion

Across the flushing strategies, the incidence of TIVAD‐related complications was very low in hospitalized patients with cancer. Within a structured, standardized, nurse‐led catheter care framework, catheter occlusion, bleeding, and catheter‐related bloodstream infections were rare, regardless of the flushing solution used. Rather than providing comparative effectiveness evidence between flushing solutions, the principal observation of this exploratory study is that uniformly low complication rates were achieved across all groups when catheter care was delivered through a consistent, protocol‐driven model. This pattern suggests that the quality and consistency of care delivery processes may contribute importantly to early catheter outcomes alongside the pharmacological choice of flushing solution; this hypothesis warrants formal testing in future studies. These observations are particularly relevant to inpatient oncology settings, where frequent catheter manipulation and high clinical workload increase the risk of early complications.

No significant differences in complication rate were observed between the heparin and normal saline groups. Importantly, given the extremely low event rate and the exploratory sample size, the absence of statistically significant differences cannot be interpreted as evidence of equivalence or non‐inferiority between flushing strategies. The uniformly low event rates observed across the groups are, however, consistent with the findings of systematic reviews reporting no clear superiority of heparin over normal saline (Wu et al. 2021; Zhong et al. 2017). Accordingly, these findings should be interpreted as hypothesis‐generating rather than as support for substituting one flushing strategy for another.

Current international infusion therapy guidelines emphasize standardized techniques and adherence to evidence‐based catheter care practices (Nickel et al. 2024). In this study, these recommendations were operationalized through nurse training and competency assessment, together with standardized pulsatile flushing, chlorhexidine antisepsis, and positive‐pressure flushing, within a protocol‐driven workflow. However, clinical practice varies considerably across health‐care settings (Vilão et al. 2024). The low complication rates observed across all three groups occurred within this standardized care model; however, the current study design does not allow the independent contribution of the care model to be determined. These findings highlight that evaluating flushing solutions in isolation, without accounting for the care delivery context, may be insufficient to explain variation in catheter outcomes. The integration of structured nurse training, protocol‐driven workflows, and standardized procedures into routine inpatient oncology care warrants further investigation as a strategy for improving vascular access management.

From an implementation perspective, the standardized care framework incorporated several components aligned with implementation science principles. These included structured training with competency assessment, biweekly observational audits using a structured checklist, feedback through routine clinical meetings, and reinforcement of standardized procedures when deviations were observed (Branch‐Elliman, Chambers, et al. 2025; Branch‐Elliman, Reyes Dassum, et al. 2025). Although implementation fidelity was not quantified using validated instruments, these nurse‐led monitoring and feedback mechanisms represent pragmatic quality assurance strategies commonly employed in real‐world clinical settings. The uniformly low complication rates observed under these conditions suggest that such implementation components may contribute to favourable catheter outcomes; however, the relative contribution of the care framework versus the flushing strategy cannot be disentangled in the current study design. Future studies should incorporate formal measures of implementation fidelity, such as adherence rates from structured checklists, and predefined implementation outcomes to better characterize the mechanisms underlying effective care delivery (Damschroder et al. 2022; Proctor et al. 2011).

The routine use of heparin in TIVAD maintenance represents a potentially modifiable clinical practice. De‐implementation science provides a framework for identifying and reducing low‐value or unnecessary interventions in health‐care systems (Grimshaw et al. 2020; Parker et al. 2022). The present findings, while exploratory and insufficient to recommend practice change, contribute preliminary data to this discourse by demonstrating that catheter‐related complications were infrequent across all flushing strategies when delivered within a standardized care framework. If confirmed in adequately powered multicenter studies, these findings could support the hypothesis that routine heparin use may be safely reduced in settings with consistently implemented standardized catheter care. Such an approach could reduce medication exposure, simplify nursing workflows, and lower costs. However, the current study does not provide sufficient evidence to recommend such a change, and any de‐implementation effort would require robust evidence from non‐inferiority or equivalence trials.

### 6.1 Implications for Nursing Practice

Although the findings of this exploratory study cannot be used to recommend specific practice changes, they carry several implications for oncology nursing. The uniformly low complication rates observed across all flushing groups underscore the potential value of structured, protocol‐driven catheter care frameworks in inpatient oncology settings and support continued emphasis on consistent implementation of standardized nursing processes, including competency‐based training, protocol adherence, and routine monitoring with feedback. The study also provides a replicable model for organizing and evaluating nurse‐led catheter care that may be adapted across institutions. More broadly, recent evidence from oncology care also supports the value of structured nurse‐led interventions and follow‐up for symptom management during systemic cancer therapy (Alqaisi et al. 2026). These preliminary data may inform nursing education and quality improvement efforts by highlighting the importance of care delivery processes, rather than pharmacological choices alone, in catheter maintenance.

### 6.2 Strengths and Limitations of the Work

This study has several strengths. First, the randomized three‐arm design allowed early catheter‐related outcomes to be observed across two saline‐based regimens and heparin lock within the same standardized care framework. Second, all randomized participants were included in the analysis, and complete outcome data were available. Third, catheter care was delivered through a structured nurse‐led protocol that included training, competency assessment, adherence monitoring, and feedback. Fourth, suspected occlusion events were adjudicated by an independent attending physician who was blinded to group allocation.

This study also has several limitations that should be considered when interpreting the findings. First, it was conducted at a single center, and the surveillance approach was changed from the originally registered in‐hospital assessment period, which allowed assessment for up to 12 weeks, to a standardized 30 days period after enrollment. This may have reduced ascertainment of later complications such as delayed thrombotic occlusion, while post‐discharge surveillance through routine outpatient records may also have missed undocumented events. However, the focus on early complications reflects the clinical priorities of inpatient oncology care, where catheter‐related events may occur during active device use. Because the assigned flushing protocol was limited to the index hospitalization and post‐discharge catheter care was not study‐mandated, variation in outpatient catheter management during the 30‐day surveillance period may also have influenced subsequent catheter‐related outcomes.

Second, the extremely low event rate, with one occlusion across 192 participants, severely limited statistical precision and precluded meaningful comparative inference between flushing strategies. The study was not designed or powered as a non‐inferiority or equivalence trial; therefore, the findings should be interpreted strictly as exploratory and hypothesis‐generating and cannot support substitution of one flushing strategy for another.

Third, although structured training, biweekly observational audits with a structured checklist, and feedback mechanisms were incorporated into the care framework, implementation fidelity and protocol adherence were not quantified using validated instruments. This makes it difficult to disentangle the relative contributions of the flushing strategy versus the standardized care processes to the observed outcomes.

Finally, the generalizability of the findings may be limited because the study was conducted in a single‐center inpatient oncology setting with a specific patient population.

### 6.3 Recommendations for Further Research

Future research should evaluate flushing strategies and nurse‐led catheter care models using adequately powered multicenter designs with longer follow‐up periods. Non‐inferiority or equivalence trials are needed before any conclusion can be drawn about substituting saline‐based flushing for heparin lock in TIVAD maintenance. Future studies should also include broader patient populations, outpatient settings, and patients with longer catheter dwell times to assess the generalizability of the findings. In addition, future research should incorporate formal measures of implementation fidelity, predefined implementation outcomes, and cost‐effectiveness analyses to clarify how standardized care processes influence catheter‐related outcomes and whether routine heparin use can be safely reduced in settings with robust protocol‐driven care.

## 7 Conclusion

This exploratory randomized study observed uniformly low TIVAD‐related complication rates in hospitalized patients with cancer across three flushing strategies when catheter care was delivered within a standardized, nurse‐led, protocol‐driven framework. These findings do not establish equivalence or non‐inferiority between flushing solutions. Instead, they generate the hypothesis that the consistency and quality of care delivery processes may be important contributors to early catheter outcomes. Optimizing catheter management in oncology practice may require attention not only to the pharmacological choice of flushing solution but also to the processes of care delivery and standardization. Multicenter studies with longer follow‐up and adequate statistical power are required to confirm these findings and evaluate long‐term efficacy, cost‐effectiveness, and generalizability across diverse health‐care settings.

## Author Contributions

**Chi‐Jia Ho:** conceptualization, investigation, writing – original draft, data curation. **Jason Chia‐Hsun Hsieh:** methodology, writing – original draft. **Hui (Grace) Xu:** writing – review and editing, formal analysis. **Chieh‐Ching Yen:** methodology, formal analysis. **Hsuan‐Chih Kuo:** resources. **Chih‐Ting Chou:** investigation. **Li‐Chin Chen:** resources. **Bo‐Cyuan Wang:** funding acquisition, writing – review and editing, validation, project administration, supervision, data curation.

## Funding

This study was supported by New Taipei City Municipal Tucheng Hospital (Built and Operated by Chang Gung Medical Foundation) (grant number: CMRPVVM0141). The funder had no role in the study design, conduct, data collection, analysis, interpretation, or manuscript preparation.

## Disclosure

Statistical statement: The authors have checked to make sure that this submission conforms, as applicable, to the Journal's statistical guidelines. There is a statistician on the author team: Jason Chia‐Hsun HSIEH. The authors affirm that the methods used in the data analyses are suitably applied to the data within the study design and context, and that the statistical findings have been implemented and interpreted correctly. The authors agree to take responsibility for ensuring that the choice of statistical approach is appropriate and is conducted and interpreted correctly as a condition of submission to the Journal.

## Ethics Statement

This study was conducted in accordance with the principles of the Declaration of Helsinki and was approved by the Institutional Review Board of Chang Gung Medical Foundation (approval number: 202101793A3).

## Consent

Written informed consent was obtained from all participants prior to enrollment.

## Conflicts of Interest

The authors declare no conflicts of interest.

## Data Availability

The data that support the findings of this study are available on request from the corresponding author. The data are not publicly available due to privacy or ethical restrictions.
